# Assessment of quality of life in people with severe and enduring anorexia nervosa: a comparison of generic and specific instruments

**DOI:** 10.1186/1471-244X-13-284

**Published:** 2013-11-07

**Authors:** Deborah Mitchison, Phillipa Hay, Scott Engel, Ross Crosby, Daniel Le Grange, Hubert Lacey, Jonathan Mond, Shameran Slewa-Younan, Stephen Touyz

**Affiliations:** 1 School of Medicine, University of Western Sydney, Sydney, Australia; 2 Centre for Health Research, School of Medicine, University of Western Sydney, Sydney, Australia; 3 School of Medicine, James Cook University, Townsville, Australia; 4 Neuropsychiatric Research Institute, Fargo, United States of America; 5 School of Medicine and Health Sciences, University of North Dakota, Grand Forks, United States of America; 6 Department of Psychiatry and Behavioral Neuroscience, University of Chicago, Chicago, United States of America; 7 Division of Population Health Sciences and Education, Eating Disorders Research Team, St George’s, University of London, London, United Kingdom; 8 School of Medicine and Public Health, University of Newcastle, Orange, Australia; 9 School of Sociology, Australian National University, Canberra, Australia; 10 School of Psychology, University of Sydney, Sydney, Australia

**Keywords:** Quality of life, Anorexia nervosa, Questionnaires, Psychometric, Eating disorder, Measurement, Burden, Treatment

## Abstract

**Background:**

Criticisms that generic measures of health-related quality of life (HRQoL) are not sensitive to impairment in anorexia nervosa (AN) has spurred the development of disease-specific measures. This study aimed to compare the psychometric properties of a generic to a disease-specific measure of HRQoL.

**Methods:**

63 participants with AN completed measures of a generic HRQoL (SF-12), disease-specific HRQoL (Eating Disorders Quality of Life Questionnaire; EDQOL), functional impairment (days out of role; DOR; Work and Social Adjustment Scale; WSAS), and eating disorder severity (Eating Disorder Examination; EDE) at baseline, post-treatment, and 6- and 12-months follow-up. Cronbach’s α was computed for the SF-12 and EDQOL (internal consistency). Correlations were assessed between SF-12/EDQOL scores and DOR, WSAS, and EDE scores (convergence validity). Three sets of three multiple linear regressions were performed using SF-12 and EDQOL scores as predictors and change in DOR, WSAS, and EDE global scores from baseline to (i) post-treatment, (ii) 6-month follow-up, (iii) and 12-month follow-up as dependent variables (predictive validity and sensitivity).

**Results:**

The EDQOL displayed stronger internal consistency (α = 0.92) than the SF-12 (α = 0.80). The SF-12 converged more strongly with DOR and the WSAS (*r*_*p*_ = −0.31 to −0.63 vs. 0.06 to 0.70), while the EDQOL converged more strongly with the EDE (*r*_*p*_ = −0.01 to 0.48 vs. -0.01 to −0.37). The SF-12 demonstrated stronger predictive validity (β = −0.55 to 0.29) and sensitivity to changes in ED severity (β = −0.47 to 0.32).

**Conclusions:**

The SF-12 is a valid and sensitive measure of HRQoL impairment in patients with AN. While the SF-12 may be preferred in research comparing EDs to other populations, and in research and practice as an indicator of functional impairment; the EDQOL may be preferred by clinicians and researchers interested in HRQoL impairment specifically associated with an ED and as an additional indicator of ED severity.

## Background

Longer duration of illness is a predictor of treatment resistance and mortality in the eating disorders (EDs) [1,2]. Treatment resistance has traditionally been defined as a lack of improvement in ED pathology following intervention. However patients who have suffered an ED for years also suffer from associated impairment in many important areas of daily life, such as in the social, work, family, and leisure domains. As such, an important end-point to consider in the treatment of enduring EDs is health-related quality of life (HRQoL) [3]. This is especially the situation for assessment of outcomes for people with severe and enduring anorexia nervosa (AN). In these patients treatment goals are often modified to emphasize improvements in social and occupational function over full weight recovery and remission of all ED symptoms [4-8].

Reviews of the relevant literature have found that all EDs, including ED not otherwise specified (EDNOS), subclinical EDs, and specific ED features are associated with impairment in HRQoL [9-11]. However, while some of this research includes an association between AN and impairment, findings have been mixed, and contention remains regarding HRQoL in this group. Although AN is regarded as the most physically and mentally debilitating of the EDs, a number of clinical and community-based studies have reported that participants with AN have less HRQoL impairment than participants with bulimia nervosa and binge eating disorder [12-14], and on some domains (e.g. social functioning) report comparable HRQoL to normal controls [13,14].

Such findings have led to a discourse regarding the accurate measurement of HRQoL in the EDs. Criticisms have been made that generic measures (including the widely used medical outcome study short forms, the SF-12 [15] and SF-36 [16]) may not be sensitive to the true level of impairment associated with EDs, or accurately differentiate between ED diagnostic groups [10,11]. Calls for the development of ED-specific instruments were made in order to increase the relevance of questions and domains of HRQoL to people with EDs, thereby increasing the overall sensitivity of HRQoL measurement. This is not a phenomenon unique to the ED field, but rather mirrored a similar disease-specific movement in other health fields, which began in cancer research [17].

The response was the development of four new instruments, three of which have been identified as having particularly strong psychometrics [18], including Engel and colleagues’ EDs quality of life (EDQOL) questionnaire [19]. The stated benefits of these ED-specific instruments include a greater sensitivity to impairment and responsiveness to change compared to generic HRQoL measures, in turn resulting in larger effect sizes in analysis. In practical terms, the greater sensitivity of these specific measures may translate into knowledge regarding the minimal change in ED symptomatology required to reflect meaningful improvement in everyday functioning.

The development and growing preference for ED-specific HRQoL measures has added to our ability to measure impairment in EDs, however there are also limitations that suggest that the use of generic instruments should not be abandoned. For one, ED specific measures cannot be used to compare EDs to other mental or physical health disorders. Furthermore, ED-specific measures attempt to measure HRQoL impairment secondary only to ED symptoms, and as such must rely on individuals’ ability to partition impairment caused by the ED versus other psychosocial problems; an endeavour complicated by the high psychological and physical comorbidity in people with EDs [20-22].

The respective strengths and limitations of generic versus specific measures of HRQoL have led to suggestions, both within and outside of the ED field, that there may be a place for using both types of measures. While this appears sensible advice, no studies to date have specifically been conducted in order to compare the relative performance of generic versus disease-specific HRQoL instruments in an ED sample. Such an investigation would provide an evidence-base that will aid the selection of instruments in future research and clinical practice.

### Aims

The current study aimed to compare the psychometrics of a disease-specific HRQoL measure (EDQOL [19]) to a generic HRQoL measure (SF-12 [15]), both commonly used in ED research and practice. The sample was made of patients with chronic AN participating in a treatment trial. This allowed for a comparison of the internal consistency, convergent validity, criterion-related validity, and sensitivity to predict change in ED pathology of the EDQoL vs. the SF-12. It also enabled the contentious issue of HRQoL and its measurement in AN to be addressed. Given our aim was more specifically to test the claims made that disease-specific measures provide a more accurate indication of HRQoL, it was hypothesized that the EDQOL would generate stronger internal consistency, criterion-related and convergent validity, and sensitivity to change than the SF-12 in this study.

## Methods

### Participants

Participants were 63 females with long-standing AN, who were randomly assigned to receive either cognitive behavioural therapy (CBT) [23] or specialist supportive clinical management (SSCM) [24]. Participants were eligible if they were female; were 18 years or older; met DSM-IV [25] criteria for AN (excluding criterion D amenorrhea, in order to align with the proposed DSM-5 changes); and had an illness duration of at least 7 years (even if there had been periods of remission). Participants were excluded from the study if they had a current manic episode or psychosis; had current alcohol or substance abuse or dependence; had a significant current medical or neurological illness, including seizure disorder (with the exception of nutrition-related alterations) that impact on weight; were currently engaged in psychotherapy and not willing to suspend this while participating in the study; or did not live within or had plans to move beyond commuting distance from the study site in the following 12 months. Both treatment arms involved 30 × 50-minute individual treatment sessions provided over a period of eight months in an outpatient setting. While treatment addressed ED symptoms, the main goal was to improve patients’ quality of life. Participants ranged in age from 20 to 62 years (*M* = 33.4, *SD* = 9.6), had a long duration of AN (*M* = 16.6 years, *SD* = 8.5), and were underweight (*M* = 16.2 body mass index (BMI; kg/m^2^), *SD* = 1.3). Most participants were diagnosed with AN restricting (vs. binge/purge) subtype (*n* = 47; 75%), were single (*n* = 36; 57%), did not have children (*n* = 50; 79%), were in full-time employment (*n* = 25; 40%) or study (*n* = 10; 16%), and had a graduate or postgraduate degree (*n* = 40; 63%).

### Measures

#### Diagnostic measures

Diagnosis at initial assessment was determined using what is generally considered the gold standard diagnostic interview for EDs, the Eating Disorder Examination (EDE) [26]. The EDE determines the frequency and severity of ED symptoms and produces four subscale scores (Restraint, Eating Concerns, Weight Concerns, and Shape Concerns) which together contribute to an overall global score. BMI and frequency of ED behaviours (objective and subjective binge eating, purging behaviours, and driven exercise) are also assessed in the EDE. The SCID-I (Structured Clinical Interview for DSM-IV-TR Axis I Disorders) [27] was also used to assess for Axis I mental disorder comorbidity.

#### Health related quality of life measures

The Medical Outcomes Study (12-item) short-form (SF-12) [15] and the Eating Disorders Quality of Life Questionnaire (EDQOL) [19] were administered to assess HRQoL. The SF-12 is a standardized generic measure, and has been used widely in research interested in the impairment associated with physiological and psychological health conditions. The 12 items contribute to 2 weighted scales, a Physical Component Summary Scale (PCS) and a Mental Component Summary Scale (MCS), each with a normative mean of 50 and standard deviation of 10. Higher scores indicate higher levels of functioning. Items on the PCS assess how health is perceived to limit everyday physical activities, how physical health is perceived to limit social functioning and productivity in work and other roles, and the extent to which pain is experienced. Items on the MCS assess how emotional health is perceived to limit social functioning and productivity in work and other roles, and the extent to which participants feel anxious, depressed, and lethargic. Strong psychometric properties have been demonstrated and norms computed for Australian population samples [15,28]. Participants who completed the SF-12 were *n* = 63 at baseline, *n* = 55 (87%) at end of treatment, *n* = 42 (67%) at 6-month follow-up, and *n* = 46 (73%) at the 12-month follow-up.

The EDQOL was designed as a disease-specific questionnaire to measure HRQoL in ED patients. It has 25 items that contribute to four subscales (Psychological, Physical/Cognitive, Work/School, and Financial), which combined produce an overall quality of life score. Each item is coded on a five-point scale and asks the participant to rate the extent to which they perceive their ED to affect their quality of life in different domains. Higher scores indicate lower ED-HRQoL. Items on the Psychological subscale assess how the ED is perceived to have impacted on thoughts and feelings about oneself; items on the Physical/Cognitive subscale assess how the ED is perceived to have impacted on physical sensations and cognitive capacity; items on the Financial subscale assess how the ED is perceived to have impacted on financial status; and items on the Work/School subscale assess how the ED is perceived to have impacted on performance at work or school. The authors have demonstrated good psychometrics for the EDQOL [19]. Participants who completed the EDQOL were *n* = 63 at baseline, *n* = 55 (87%) at end of treatment, *n* = 43 (68%) at 6-month follow-up, and *n* = 48 (76%) at the 12-month follow-up.

#### Measures to assess validity

#### Convergent validity

Convergent validity refers to the extent that measures of the same or similar theoretical construct are related to each other. The Work and Social Adjustment Scale [WSAS; 29] and days out of role (DOR) question were chosen as indicators of concurrent convergent validity for the SF-12 and EDQOL. The WSAS is a short five-item measure of disease-specific functional impairment in the domains of work, home duties, social leisure, private leisure, and close relationships. It has acceptable psychometric properties [29]. The DOR question was modelled on questions employed in the American National Comorbidity Survey [30]. Specifically, participants were asked: “*During the past four weeks, on how many days, if any, were you unable to complete your work, study or household responsibilities because of any problem with your (physical or emotional) health?*” A response between 0 (no days) and 28 (every day) was required. Research has indicated correlations in the order of −0.40 to −0.50 between the DOR question and scores on the PCS and MCS of the SF-12 [31]. The global scale and subscales of the EDE (described above) was also used to indicate convergence with ED severity, with the underlying assumption that poorer HRQoL should be associated with greater pathology.

#### Predictive criterion-related validity

Predictive criterion-related validity refers to the ability of scores on a given measure to accurately predict a future outcome (the criterion). In the present study, the predictive validity of the SF-12 and EDQOL administered at baseline was tested against the criterion of change in DOR and WSAS scores from baseline to post-treatment.

#### Sensitivity to change in eating disorder pathology

The sensitivity of a measure refers to the extent to which changes in scores of the measure predicts changes in another measure. To assess the sensitivity of the SF-12 and EDQOL to changes in ED pathology, the ability of the SF-12 and EDQOL to predict baseline to post-treatment changes in the EDE global score was assessed.

### Procedure

The study was approved by the Human Research Ethics Committee of the University of Sydney (Protocol No: 9669). Participants were recruited from July 2007 through November 2010 by advertising to clinicians, clinics treating people with EDs, and on generic websites. After telephone screening (*N* = 159) to determine eligibility, 73 (46%) eligible participants were invited for in-person assessment. Respective site study coordinators described the protocol in detail to these eligible participants before written informed consent was obtained and the assessments conducted. Eighty-six percent (*N* = 63) of eligible participants agreed to randomization. Participants were assigned to and received either CBT or SSCM. In addition to the pre-treatment assessment, participants were assessed immediately post-treatment, and 6- and 12-months following the end of treatment. Assessments were conducted by trained psychologists blind to treatment assignment, and at a place of convenience to the participant which was not the place of treatment.

### Data analysis

The Statistical Package for the Social Sciences (SPSS) version 20.0 was used to carry out analyses. Descriptive statistics of baseline demographic information were computed, and compared between the CBT and SSCM groups using student *t*-tests (age, BMI, duration of illness) and chi-square tests (AN subtype, relationship status, employment/study status, children status, highest education level achieved). Given no significant differences were observed on any of these variables (all *p* > 0.05), the CBT and SSCM groups were grouped together in subsequent baseline analyses. In analyses that used post-treatment data, treatment assignment was entered as a covariate.

To assess internal consistency, Cronbach’s α was computed on the baseline data for the subscale and total scores of the SF-12 and EDQOL. Validity and sensitivity analyses were run with and without DSM-IV-TR Axis I comorbidity as a covariate. Pearson product–moment correlations (*r*_*p*_) were computed to facilitate validity analyses. To assess concurrent convergent validity, baseline WSAS and DOR scores were correlated with baseline SF-12 and EDQOL subscale scores. To assess concurrent convergence with ED severity, the baseline EDE subscale and global scores were correlated with the baseline SF-12 and EDQOL subscale scores. To assess predictive criterion-related validity, incremental change scores from baseline to post-treatment for the DOR and WSAS were computed, and then regressed against baseline SF-12 and EDQOL subscale scores. To assess the sensitivity to predict change in ED severity, incremental change in the EDE global score from baseline to post-treatment was computed and regressed against baseline SF-12 and EDQOL subscale scores. Analyses were considered significant at *p* < 0.05. Post-hoc power analyses using the PASS 11 software [32] revealed adequate power (0.8) to detect medium-sized correlations for the convergence validity analyses, and *R*^*2*^ coefficients in the order of 0.28 - 0.35 for the predictive validity and sensitivity analyses.

## Results

Treatment effects for the randomized controlled trial, within which the current study is embedded, have been analysed and are reported elsewhere [8].

### Internal consistency

Cronbach’s α for the total EDQOL scale was 0.92 and for the total SF-12 scale was 0.80. For the EDQOL subscales the α-coefficients were: 0.91 (Psychological), 0.86 (Physical/Cognitive), 0.73 (Work/School), and 0.81 (Financial). For the SF-12 summary scales the α-coefficients were: 0.71 (PCS) and 0.80 (MCS).

### Validity

#### Convergence with functional impairment

The Pearson Product Moment correlations to establish convergent validity are presented in Table 1. Most correlations between the SF-12/EDQOL subscales and the WSAS scale and DOR question were significant, and ranged from moderate to strong (*r*_*p*_ > 0.30), indicating convergence with measures of functional impairment. Correlations for the SF-12 summary scales with the WSAS and DOR ranged between −0.31 to −0.63. Correlations for the EDQOL subscales with the WSAS and DOR ranged between 0.06 to 0.70. When partial correlations were computed, controlling for Axis I comorbidity, the strength of these correlations reduced (see Table 1).

**Table 1:** Convergent validity of the SF-12 and EDQOL

	**SF-12 PCS**	**SF-12 MCS**	**EDQOL psychological**	**EDQOL physical/cognitive**	**EDQOL financial**	**EDQOL work/school**
***No Covariates***						
WSAS	−0.36**	−0.63**	0.70**	0.66**	0.29*	0.59**
DOR	−0.31*	−0.35**	0.19	0.53**	0.06	0.51**
EDE-G	−0.01	−0.37**	0.48**	0.41**	0.15	0.42**
EDE-R	0.12	−0.31**	0.41**	0.28*	0.16	0.24
EDE-EC	−0.23	−0.33**	0.46**	0.45**	0.19	0.42**
EDE-WC	0.03	−0.36**	0.40**	0.33**	−0.01	0.38**
EDE-SC	0.01	−0.25*	0.35**	0.31*	0.17	0.39**
***Covarying for Axis I Comorbidity***						
WSAS	−0.25	−0.43**	0.57**	0.52**	0.28*	0.51**
DOR	−0.25	−0.08	0.14	0.45**	−0.08	0.31*
EDE-G	0.11	−0.05	0.25	0.20	−0.05	0.18
EDE-R	0.21	−0.13	0.26	0.12	0.03	0.08
EDE-EC	−0.10	−0.09	0.31*	0.29*	0.05	0.23
EDE-WC	0.13	0.04	0.09	0.09	−0.22	0.08
EDE-SC	0.07	0.03	0.14	0.16	−0.02	0.19

**p < 0.01, *p < 0.05; SF-12 = Medical Outcomes Study Short Form; PCS = Physical Component Summary Scale; MCS = Mental Component Summary Scale; EDQOL = Eating Disorders Quality of Life Questionnaire; WSAS = Work and Social Adjustment Scale; DOR = days out of role; EDE = Eating Disorders Examination; R = Restraint subscale; EC = Eating Concerns subscale; WC = Weight Concerns subscale; SC = Shape Concerns subscale; G = Global scale.

#### Convergence with eating disorder severity

Table 1 also contains the correlations of the SF-12/EDQOL with the EDE global and subscales. These correlations were also mostly significant and moderate to strong, indicating convergence with a measure of ED severity. The correlations for the SF-12 summary scales with the EDE global scale and subscales ranged between 0.01 to −0.37. The correlations for the EDQOL subscales with the EDE global and subscales ranged between −0.01 to 0.48. In order to assess for any effect of age on convergence with ED severity, the analyses were re-run as partial correlations controlling for age. No effect of age was found. However partial correlations controlling for Axis I comorbidity resulted in a loss of almost all significant correlations (see Table 1).

#### Predictive criterion-related validity

The SF-12 and EDQOL subscales were entered as predictors into multiple linear regressions (MLRs) with change in DOR (*n* = 45) and WSAS (*n* = 46) from baseline to post-treatment as the dependent variables, for participants with complete data (see Table 2). The MCS of the SF-12 emerged as the only significant independent predictor of both change in DOR (*r*_*p*_ = −0.31, *p* = 0.02) and change in the WSAS (*r*_*p*_ = −0.55, *p* < 0.001) scores by post-treatment. Table 2 also shows post-hoc MLR analyses that were run on the data for the two follow-up time points at 6-months and 12-months post treatment. The MCS of the SF-12 remained the sole predictor of change in WSAS scores at both the 6-month (*r*_*p*_ = −0.51, *p* < 0.001) and 12-month (*r*_*p*_ = −0.44, *p* = 0.00) follow-up. The Work/School (*r*_*p*_ = 0.31, *p* = 0.03) and Financial (*r*_*p*_ = −0.21, *p* = 0.11) EDQOL subscales predicted change in DOR by the 6-month follow-up and the Work/School subscale remained a significant predictor of change in DOR after 12 months (*r*_*p*_ = 0.29, *p* = 0.04). When these analyses included Axis I comorbidity as a covariate, few differences emerged (see Table 2): the MCS of the SF-12 no longer predicted change in DOR immediately post-treatment, whereas the Financial subscale of the EDQOL did; and there were no significant predictors of change in DOR by the 12-month follow-up.

**Table 2:** Multiple linear regression models using the SF-12 and EDQOL as predictors of change in functional impairment (predictive validity)

**Dependent**	**Significant predictors**	**B**	**SE (B)**	***β***	***t***	***p***
***Without Axis I Comorbidity as a covariate***						
DOR change: baseline to post-treatment	SF-12 MCS	−0.23	0.11	−0.31	−2.10	0.04
*R*^*2*^_*adj*_ = 0.07						
DOR change: baseline to 6-month follow-up	EDQOL Work/School	5.20	2.07	0.40	2.51	0.02
	EDQOL Financial	−4.18	2.12	−0.31	−1.97	0.06
*R*^*2*^_*adj*_ = 0.14						
DOR change: baseline to 12-month follow-up	EDQOL Work/School	2.71	1.49	0.29	1.82	0.08
*R*^*2*^_*adj*_ = 0.06						
WSAS change: baseline to post-treatment	SF-12 MCS	−0.43	0.10	−0.55	−4.34	<0.001
*R*^*2*^_*adj*_ = 0.28						
WSAS change: baseline to 6-month follow-up	SF-12 MCS	−0.38	0.10	−0.51	−3.61	0.001
*R*^*2*^_*adj*_ = 0.24						
WSAS change: baseline to 12-month follow-up	SF-12 MCS	−0.33	0.11	−0.44	−3.00	0.01
*R*^*2*^_*adj*_ = 0.17						
***With Axis I Comorbidity as a Covariate***						
DOR change: baseline to post-treatment	Axis I Comorbidities	6.97	2.62	0.40	2.67	0.01
	EDQOL Financial	−4.29	2.06	−0.31	−2.08	0.04
*R*^*2*^_*adj*_ = 0.14						
DOR change: baseline to 6-month follow-up	EDQOL Work/School	4.91	2.33	0.35	2.11	0.04
	EDQOL Financial	−4.36	2.16	−0.34	−2.02	0.05
*R*^*2*^_*adj*_ = 0.12						
DOR change: baseline to 12-month follow-up	-	-	-	-	-	-
*R*^*2*^_*adj*_ = 0.00						
WSAS change: baseline to post-treatment	SF-12 MCS	−0.41	0.10	−0.52	−3.96	<0.001
*R*^*2*^_*adj*_ = 0.25						
WSAS change: baseline to 6-month follow-up	SF-12 MCS	−0.36	0.11	−0.48	−3.23	0.00
*R*^*2*^_*adj*_ = 0.21						
WSAS change: baseline to 12-month follow-up	SF-12 MCS	−0.34	0.12	−0.43	−2.85	0.01
*R*^*2*^_*adj*_ = 0.16						

*R*^*2*^_*adj*_ = variance in the dependent variable accounted for by the predictor variable(s), adjusting for statistical shrinkage; SF-12 = Medical Outcomes Study Short Form; PCS = Physical Component Summary Scale; EDQOL = Eating Disorders Quality of Life Questionnaire; DOR = days out of role; WSAS = Work and Social Adjustment Scale.

### Sensitivity to change in eating disorder pathology

A MLR was conducted with change in the global EDE score from baseline to post-treatment as the dependent variable (see Table 3), and using data from participants with complete data (*n* = 45). Baseline scores on the MCS of the SF-12 (*r*_*p*_ = −0.34, *p* = 0.01) emerged as the strongest independent predictor, followed by treatment assignment (*r*_*p*_ = −0.25, *p* = 0.05). Post-hoc analyses of the follow-up data revealed that the MCS of the SF-12 (*r*_*p*_ = −0.24, *p* = 0.07) and treatment assignment (*r*_*p*_ = −0.23, *p* = 0.08) remained significant predictors of change in the global EDE scale from baseline to 6-month follow-up (*n* = 38), while the PCS of the SF-12 (*r*_*p*_ = 0.32, *p* = 0.02) emerged as the sole independent predictor of change in the EDE global score after 12 months (*n* = 41, see Table 3). These variables remained significant predictors when analyses included Axis I comorbidity as a covariate (see Table 3).

**Table 3:** Multiple linear regression models using the SF-12 and EDQOL subscales as predictors of change in eating disorder pathology

**Dependent**	**Significant predictors**	**B**	**SE (B)**	***β***	***t***	***p***
***Without Axis I Comorbidity as a Covariate***						
EDE change: baseline to post-treatment	SF-12 MCS	−0.05	0.01	−0.47	−3.36	0.002
	Treatment assignment	−1.02	0.35	−0.40	−2.88	0.006
*R*^*2*^_*adj*_ = 0.23						
EDE change: baseline to 6-month follow-up	SF-12 MCS	−0.04	0.02	−0.36	−2.20	0.04
	Treatment assignment	−0.86	0.40	−0.35	−2.13	0.04
*R*^*2*^_*adj*_ = 0.12						
EDE change: baseline to 12-month follow-up	SF-12 PCS	0.05	0.02	0.32	2.11	0.04
*R*^*2*^_*adj*_ = 0.08						
***With Axis I Comorbidity as a Covariate***						
EDE change: baseline to post-treatment	SF-12 MCS	−0.04	0.01	−0.43	−2.85	0.01
	Treatment assignment	−0.84	0.36	−0.35	−2.31	0.03
*R*^*2*^_*adj*_ = 0.16						
EDE change: baseline to 6-month follow-up	Treatment assignment	−0.81	0.44	−0.33	−1.87	0.07
	SF-12 MCS	−0.03	0.02	−0.32	−1.82	0.08
*R*^*2*^_*adj*_ = 0.08						
EDE change: baseline to 12-month follow-up	SF-12 PCS	0.05	0.03	0.33	2.11	0.04
*R*^*2*^_*adj*_ = 0.08						

*R*^*2*^_*adj*_ = variance in the dependent variable accounted for by the predictor variable(s), adjusting for statistical shrinkage; SF-12 = Medical Outcomes Study Short Form; PCS = Physical Component Summary Scale; EDQOL = Eating Disorders Quality of Life Questionnaire; EDE = Eating Disorders Examination (global scale).

## Discussion

The aim of this study was to examine and compare the psychometric properties of a generic measure (the SF-12) to a disease-specific measure (the EDQOL) of HRQoL in a clinical sample of patients with AN. It was hypothesised that the EDQOL would out-perform the SF-12 in terms of its reliability, validity, and sensitivity to predict changes in ED pathology. This hypothesis was only partially supported. Consistent with the hypothesis, the EDQOL demonstrated stronger internal consistency than the SF-12 and also converged more consistently with indicators of ED severity. In contrast, the SF-12 converged more consistently with indicators of functional impairment, and was better able to predict changes in both functional impairment and ED severity across time.

The development of ED-specific instruments for HRQoL was in part spurred by a concern that generic measures were not sufficiently sensitive to impairment in the EDs, and particularly in AN. The current study has assessed HRQoL using both a generic and an ED-specific instrument in a sample diagnosed with AN. Contrary to our hypotheses, the generic measure of HRQoL – the SF-12 – was found to be more predictive of changes not only in functional impairment but also in ED pathology, compared to the disease-specific EDQOL. Thus this study has challenged previous assertions by demonstrating that a generic measure of HRQoL is valid and sensitive to pathology in a clinical ED sample.

The SF-12 may also be a more accurate measure of HRQoL than the EDQOL, as it showed stronger convergence with the WSAS and DOR question. All subscales of the SF-12, but not of the EDQOL, were significantly correlated with the WSAS scale and the DOR question. The WSAS and DOR question are indicators of functional impairment, a construct closely and theoretically aligned to HRQoL. When functional impairment (including the ability to attend work) is a result of poor health, as is often the case in patients with AN, then it would be expected to be highly correlated with HRQoL. Due to its personal and broad societal impacts functional impairment has important public health implications, and as such measures that are able to capture functional impairment contribute to a greater understanding of the overall burden of EDs. On the other hand, the EDQOL had stronger convergence with ED severity. All EDQOL subscales (except Financial) had significant correlations with the EDE scales, whereas only the MCS of the SF-12 was correlated with the EDE. As such, the EDQOL can be helpful in understanding the severity of an ED, given that high EDQOL scores are closely associated with high EDE scores. The relative convergence of the SF-12 and EDQOL with functional impairment versus ED pathology may be expected based on the specificity of the instruments. The generic nature of the SF-12 allows it to assess overall impairment in functioning - not only that perceived to stem from an ED - and as such is expected to align with other generic measures of impairment. Similarly, because the EDQOL was designed for people with EDs, it may be expected to align more closely with ED measures. Interestingly, once Axis I comorbidity was controlled for, many of the correlations with measures of functional impairment and ED severity were lost. This may signify that comorbid disorders accounted for much of the association with impairment and severity. It may also indicate that with greater ED severity the likelihood of having a comorbid disorder increases, and that this is associated with overall greater impairment.

The MCS of the SF-12 was consistently predictive of scores on the WSAS following treatment. However while the MCS was also predictive of DOR immediately post-treatment, it was not so at the follow-up time-points, 6 and 12 months post-treatment. Rather, the EDQOL (specifically the Work/School and Financial subscale) emerged as a significant predictor of DOR 6 and 12 months following treatment. In interpreting these findings, poorer mental wellbeing and functioning in work or school prior to treatment was associated with greater improvements in the occupational, academic, household, and interpersonal domains post-treatment. This likely demonstrates that those who are functioning less well prior to treatment have more room to improve, and this is able to be reflected particularly well by the MCS of the SF-12, but also to some extent on the Work/School and Financial subscales of the EDQOL.

A limitation of the current study may have been the use of the SF-12 rather than the longer scale from which it was derived, the SF-36, which may be more comparable to the EDQOL in design. A more balanced comparison of generic vs. disease-specific instruments may have used the EDQOL and SF-36, both of which are similar in length and have multiple subscales. Further, the SF-36 may have stronger psychometric properties than the SF-12 [33]. Test-retest reliability is an important psychometric indicator that we were unable to assess in the current study. Although this could have been achieved by adding another pre-treatment assessment point, this was decided against in order to limit participant burden in the randomized controlled trial in which the current study was embedded. Another limitation with the design of this study is that it did not allow for comparisons with other diagnostic subgroups or healthy controls. These comparisons would be of interest in future research since several previous studies have found that compared to people with other ED diagnoses or in the general community, people with AN demonstrate less or commensurate impairment in HRQoL [12-14]. The omission of amenorrhea as a criterion for AN may be seen as a limitation. This was done in order to be more consistent with the proposed DSM-5 criteria for AN and as such to have greater relevance to future studies using these criteria. It is acknowledged however that this may have resulted in a sample that differed from previous studies’ samples based on DSM-IV criteria. Participants in the current study may also have been older than those in previous AN studies. This was influenced by the aims of the treatment trial, which was to assess treatment efficacy in participants with long-standing AN. Finally, it is important to emphasise that our findings relate specifically to the population of chronic AN, and that although our findings are possibly relevant to other populations, this would need to be verified by future research.

In regards to advice for researchers and practitioners who wish to measure HRQoL, the decision ultimately comes down to the design and purpose of measurement. Should cross-comparisons be desired with the general population or other diagnostic groups, then generic measures, such as the SF-12 or SF-36 should be applied. However, the implication from this study is that if the sample of interest is AN-only then either the SF-12 or EDQOL could be used. If users require a measure that will also provide an indication of ED severity, then the EDQOL could be relatively more useful. Conversely, if the aim is to provide an indication of functional impairment, then the SF-12 may be the preferred option. On the other hand, and in line with previous suggestions, if time and resources permit, generic and disease-specific measures may be used in tandem.

## Conclusion

Our findings indicate that the EDQOL, a disease-specific measure of HRQoL, and the SF-12, a generic measure of HRQoL, are reliable and valid measures; and that the SF-12 is also sensitive to changes in pathology in a sample of patients with enduring AN. This provides evidence against previous assertions that generic measures of HRQoL are not sufficiently sensitive to impairment associated with AN. Both the SF-12 and the EDQOL are useful measures of HRQoL for practitioners and researchers who work with patients with AN.

## Abbreviations

HRQoL: Health-related quality of life; ED: Eating disorder; AN: Anorexia nervosa; EDQOL: Eating disorder quality of life questionnaire; SF-12: Medical Outcomes Study (12-item) Short-Form; CBT: Cognitive behavioural therapy; SSCM: Specialist Supportive Clinical Management; BMI: Body mass index; EDE(−Q): Eating Disorder Examination (Questionnaire); PCS: Physical component summary scale; MCS: Mental component summary scale; WSAS: Work and social adjustment scale; DOR: Days out of role; SPSS: Statistical package for the social sciences; MLR: Multiple linear regressio.

## Competing interests

The authors declare that they have no competing interests.

## Authors’ contributions

DM Design, methods, analysis, interpretation. PH Design, methods, interpretation. SE Design, interpretation and manuscript. RC Interpretation, analytic methods and manuscript. DLG Interpretation and manuscript. HL Interpretation and manuscript. JM Interpretation and manuscript. SSY Interpretation and manuscript, ST Responsible for data collection, larger study, funding, interpretation. All authors read and approved the final manuscript.

## Pre-publication history

The pre-publication history for this paper can be accessed here:

http://www.biomedcentral.com/1471-244X/13/284/prepub

## References

[B1] Reas DL Williamson DA Martin CK Zucker NL Duration of illness predicts outcome for bulimia nervosa: A long-term follow-up study Int J Eat Disord 2000 27 4 428 434 10.1002/(SICI)1098-108X(200005)27:4<428::AID-EAT7>3.0.CO;2-Y 10744849

[B2] Keel PK Dorer D Eddy K Franko D Charatan D Herzog D PRedictors of mortality in eating disorders Arch Gen Psychiatry 2003 60 2 179 183 10.1001/archpsyc.60.2.179 12578435

[B3] Coelho R Ramos S Prata J Bettencourt P Ferreira A Cerqueira-Gomes M Heart failure and health related quality of life Clinical practice and epidemiology in mental health 2005 1 19 doi:10.1186/1745-0179-1-19 10.1186/1745-0179-1-19 PMC1274338 16202163

[B4] Touyz S Le Grange D Lacey H A randomised control trial of nonspecific supportive clinical management (NSCM) versus cognitive behaviour therapy (CBT) in longstanding anorexia nervosa Aust N Z J Psychiatry 2009 43 Suppl. 1: A13

[B5] Williams KD Dobney T Geller J Setting the eating disorder aside: An alternative model of care Eur Eat Disord Rev 2010 18 2 90 96 10.1002/erv.989 20099264

[B6] Strober M Grilo CM, Mitchell JE The chronically ill patient with anorexia nervosa: Development, phenomenology, and therapeutic considerations The treatment of eating disorders: A clinical handbook 2010 London: The Guilford Press 225 238

[B7] Hay PJ Touyz S Sud R Treatment for severe and enduring anorexia nervosa: a review Aust N Z J Psychiatry 2012 46 12 1136 1144 10.1177/0004867412450469 22696548

[B8] Touyz S Le Grange D Lacey H Hay P Smith R Maguire S Bamford B Pike KM Crosby RD Treating Severe and Enduring Anorexia Nervosa: A Randomized Control Trial Psychol Med 2013 in press 10.1017/S0033291713000949 23642330

[B9] Jenkins PE Hoste RR Meyer C Blissett JM Eating disorders and quality of life: A review of the literature Clin Psychol Rev 2011 31 1 113 121 10.1016/j.cpr.2010.08.003 20817335

[B10] Hay PJ Mond J How to ’count the cost’ and measure burden? A review of health-related quality of life in people with eating disorders J Ment Health 2005 14 6 539 552 10.1080/09638230500400274

[B11] Engel SG Adair CE Hayas CL Abraham S Health-related quality of life and eating disorders: A review and update Int J Eat Disord 2009 42 2 179 187 10.1002/eat.20602 18949768

[B12] Padierna A Quintana J Arostegui I Gonzalez N Horcajo M The health-related quality of life in eating disorders Qual Life Res 2000 9 6 667 674 10.1023/A:1008973106611 11236856

[B13] Mond JM Hay P Rodgers B Owen C Beumont P Assessing quality of life in eating disorder patients Quality of Life Research: An International Journal of Quality of Life Aspects of Treatment, Care & Rehabilitation 2005 14 1 171 178 10.1007/s11136-004-2657-y 15789951

[B14] Doll HA Petersen SE Stewart-Brown SL Eating disorders and emotional and physical well-being: Associations between student self-reports of eating disorders and quality of life as measured by the SF-36 Quality of Life Research: An International Journal of Quality of Life Aspects of Treatment, Care & Rehabilitation 2005 14 3 705 717 10.1007/s11136-004-0792-0 16022064

[B15] Ware JEJ Kosinski M Keller SD A 12-Item Short-Form Health Survey: Construction of Scales and Preliminary Tests of Reliability and Validity Medical Care 1996 34 3 220 233 10.1097/00005650-199603000-00003 8628042

[B16] Ware J Kosinski M Keller S SF-36 Physical and Mental Health Summary Scales: A User’s Manual 1994 Boston: The Health Institute

[B17] Bowling A Measuring disease: A review of disease-specific quality of life measurement scales 2001 2 Buckingham: Open University Press

[B18] Tirico PP Stefano SC Blay SL Validity studies of quality of life instruments for eating disorders: systematic review of the literature J Nerv Ment Dis 2010 198 12 854 859 10.1097/NMD.0b013e3181fe7294 21135634

[B19] Engel SG Wittrock DA Crosby RD Wonderlich SA Mitchell JE Kolotkin RL Development and Psychometric Validation of an Eating Disorder-Specific Health-Related Quality of Life Instrument Int J Eat Disord 2006 39 1 62 71 10.1002/eat.20200 16345055

[B20] Hudson JI Hiripi E Pope HG Jr Kessler RC The Prevalence and Correlates of Eating Disorders in the National Comorbidity Survey Replication Biol Psychiatry 2007 61 3 348 358 10.1016/j.biopsych.2006.03.040 16815322 PMC1892232

[B21] Johnson J Cohen P Kasen S Brook J Eating disorders during adolescence and the risk for physical and mental disorders during early adulthood Arch Gen Psychiatry 2002 59 6 545 552 10.1001/archpsyc.59.6.545 12044197

[B22] Mond J Rodgers B Hay P Korten A Owen C Beumont P Disability associated with community cases of commonly occurring eatinq disorders Aust N Z J Public Health 2004 28 3 246 251 10.1111/j.1467-842X.2004.tb00703.x 15707171

[B23] Pike KM Walsh BT Vitousek K Wilson GT Bauer J Cognitive behavior therapy in the posthospitalization treatment of anorexia nervosa Am J Psychiatry 2003 160 11 2046 2049 10.1176/appi.ajp.160.11.2046 14594754

[B24] McIntosh VVW Jordan J Luty SE Carter FA McKenzie JM Bulik CM Joyce PR Specialist supportive clinical management for anorexia nervosa Int J Eat Disord 2006 39 8 625 632 10.1002/eat.20297 16937382

[B25] (APA) APA Diagnostic and Statistical Manual for Mental Disorders Fourth Edition, Text Revision edition 2000 Washington DC: American Psychiatric Association

[B26] Fairburn CG Cooper Z Fairburn CG, Wilson G The Eating Disorder Examination Binge Eating: Nature, Assessment and Treatment 1993 12 New York: Guildford Press

[B27] First MB Gibbon M Hilsenroth MJ, Segal DL The Structured Clinical Interview for DSM-IV Axis I Disorders (SCID-I) and the Structured Clinical Interview for DSM-IV Axis II Disorders (SCID-II) Comprehensive handbook of psychological assessment, Vol 2: Personality assessment 2004 Hoboken, NJ US: John Wiley & Sons Inc 134 143

[B28] Sanderson K Andrews G The SF-12 in the Australian population: cross-validation of item selection Aust N Z J Public Health 2002 26 4 343 345 10.1111/j.1467-842X.2002.tb00182.x 12233955

[B29] Mundt JC Marks IM Shear MK Greist JM The Work and Social Adjustment Scale: a simple measure of impairment in functioning Br J Psychiatry 2002 180 5 461 464 10.1192/bjp.180.5.461 11983645

[B30] Kessler RC Frank RG The impact of psychiatric disorders on work loss days Psychol Med 1997 27 4 861 873 10.1017/S0033291797004807 9234464

[B31] Mond JM Hay PJ Rodgers B Owen C Recurrent binge eating with and without the ’undue influence of weight or shape on self-evaluation’: Implications for the diagnosis of binge eating disorder Behav Res Ther 2007 45 5 929 938 10.1016/j.brat.2006.08.011 17010307

[B32] Hintze J PASS 11 2011 LLC, Kaysville, Utah, USA: NCSS

[B33] Schofield MJ Mishra G Validity of the SF-12 compared with the SF-36 Health Survey in pilot studies of the Australian Longitudinal Study on Women’s Health J Health Psychol 1998 3 2 259 271 10.1177/135910539800300209 22021364

